# Supplementary material for: IL-6/STAT3 pathway induced deficiency of RFX1 contributes to Th17-dependent autoimmune diseases via epigenetic regulation
Source: Nat Commun. 2018 Feb 8;9:583. doi: 10.1038/s41467-018-02890-0 (PMC5805701; doi:10.1038/s41467-018-02890-0)
Supplement: Supplementary file 1 — Supplementary Information [file 41467_2018_2890_MOESM1_ESM.pdf]

IL-6/STAT3 pathway induced deficiency of RFX1 contributes to Th17-dependent autoimmune diseases via epigenetic regulation

Zhao et al.

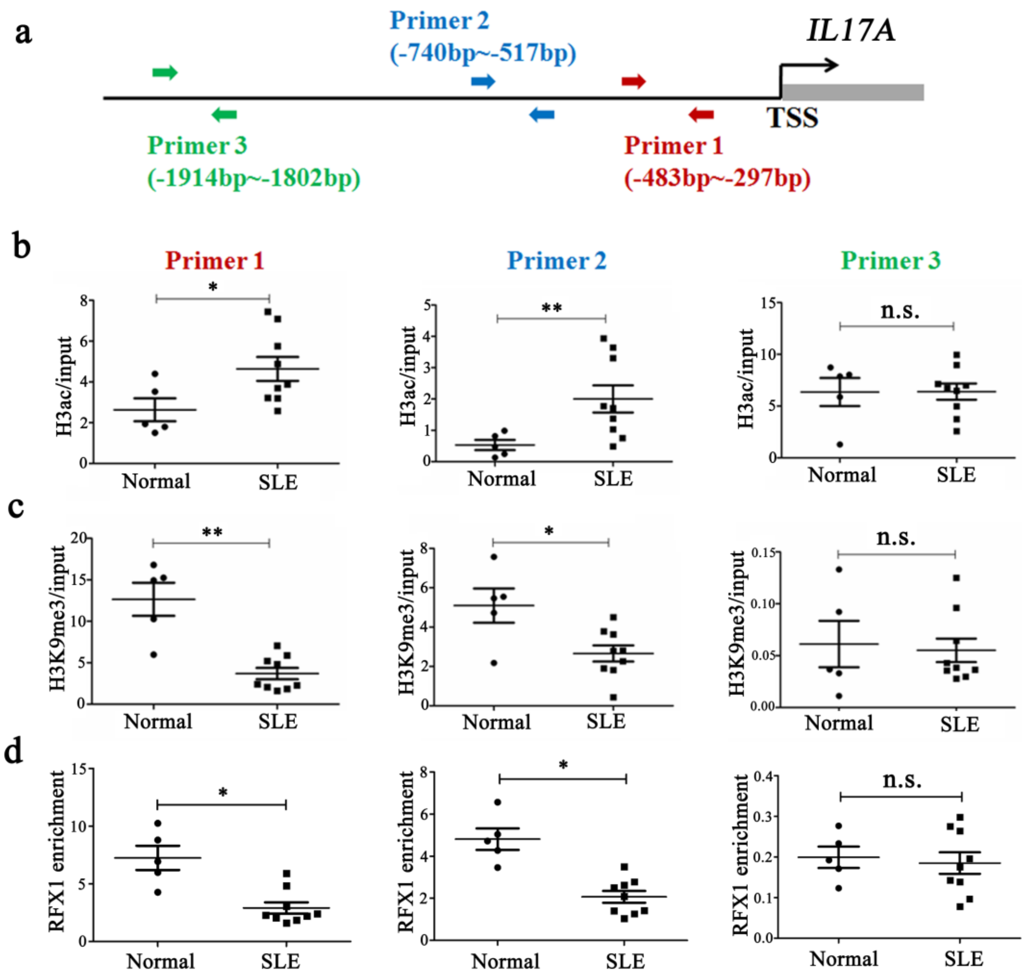

Supplementary Figure 1 The levels of histone H3 acetylation, H3K9 tri-methylation and RFX1 enrichment in the promoter of *IL17A* in CD4^{+} T cells of SLE patients (n=9) and normal controls (n=5). (a) The locations of three pairs of primers for ChIP-qPCR. Primer 1 and Primer 2 are close to the RFX1 binding sites, whereas Primer 3 is far away from the RFX1 binding site. **(b-d)** ChIP-qPCR analysis of H3 acetylation levels **(b)**, H3K9 tri-methylation levels **(c)** and RFX1 enrichment levels **(d)** in the promoter region 2000 bp upstream of the transcription start site (TSS) of the *IL17A* gene. Small horizontal lines indicate the mean (±s.e.m.) in the (**b**), (**c**) and (**d**). *P<0.05 and **P<0.01, compared between the indicated groups. n.s., not significant. P-values were determined using two-tailed Student's *t*-tests and Mann-Whitney U test.

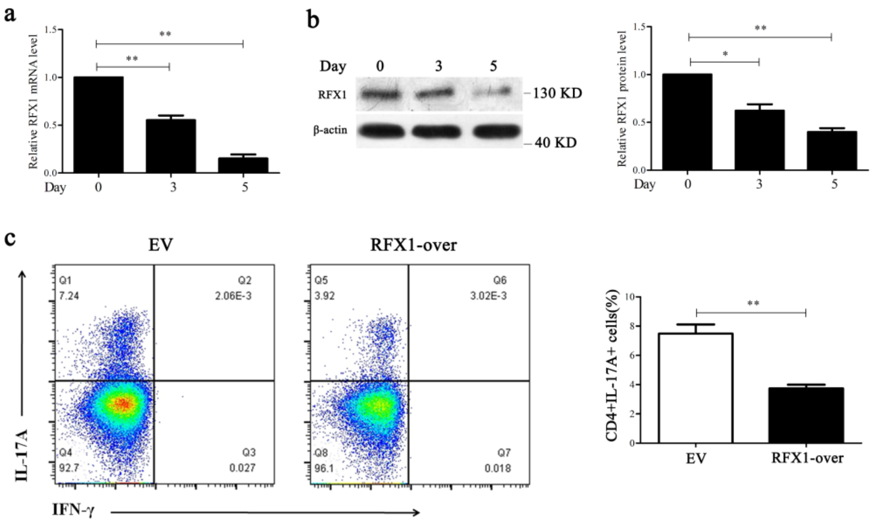

Supplementary Figure 2 RFX1 regulates human Th17 differentiation in vitro. (a) qPCR analysis of RFX1 expression in induced Th17 cells polarized with Th17-polarizing conditions on day 3 and day 5; results are presented relative to RFX1 expression in naïve T cells. **(b)** Representative western blot of RFX1 protein in induced Th17 cells on day 3 and day 5; results are presented relative to RFX1 expression in naïve T cells. **(c)** Representative flow cytometry plots of $CD4^{+}IL\text{-}17A^{+}$T cells in induced Th17 cells on day 3 post-transfection with RFX1 expression vector (RFX1-over) or empty expression vector (EV). Numbers adjacent to outlined areas indicate the percentage of cells in each. Data are representative of three independent experiments (mean ± s.d.; n=3). *P<0.05 and **P<0.01, compared between the indicated groups. P-values were determined using two-tailed Student's *t*-tests.

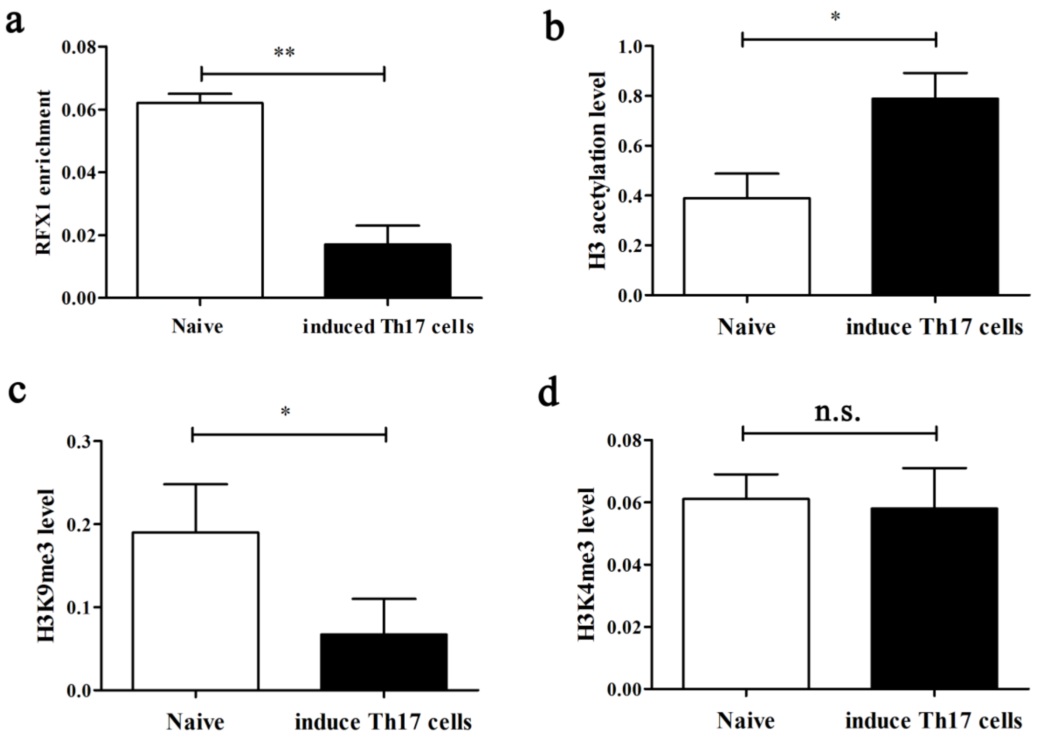

Supplementary Figure 3 The levels of RFX1 enrichment, histone H3 acetylation, H3K9 tri-methylation and H3K4 tri-methylation in the promoter of *IL17A*. (a-d) ChIP-qPCR analysis of the levels of RFX1 enrichment, H3 acetylation, H3K9me3 and H3K4me3 in the promoter of *IL17A* in the induced Th17 cells, relative to the levels in naive T cells. Data are representative of three independent experiments (mean ± s.d.; n=3). *P<0.05 and **P<0.01, compared with the indicated groups. n.s., not significant. P-values were determined using two-tailed Student's *t*-tests.

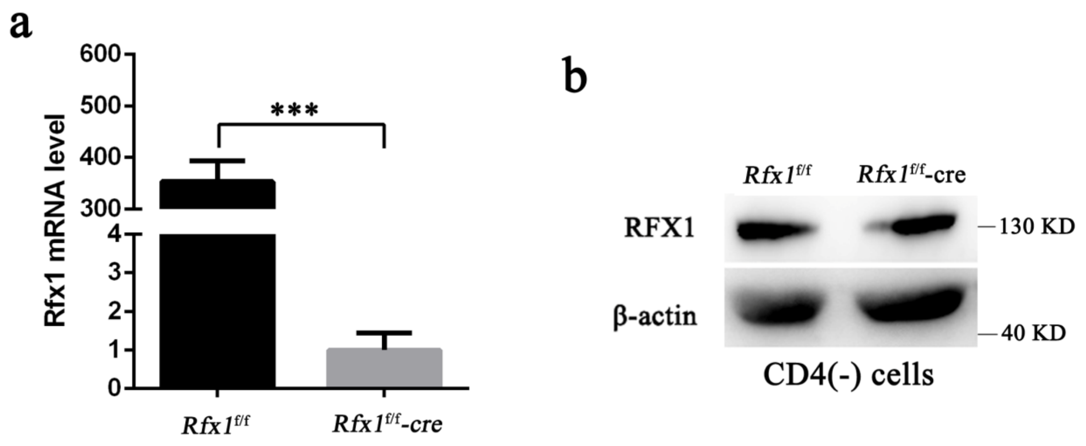

Supplementary Figure 4 Rfx1 expression in conditional knockout mice. (a) qPCR analysis of the relative mRNA expression level of Rfx1 in $CD4^{+}$ T cells from *Rfx1*$^{f/f}$ and *Rfx1*$^{f/f}$-cre mice. **(b)** Representative western blot for Rfx1 protein in $CD4^{(-)}$ T cells from *Rfx1*$^{f/f}$ and *Rfx1*$^{f/f}$-cre mice. Data are representative of three independent experiments (mean ± s.d.; n=3). ***P<0.001, compared between the indicated groups. P-values were determined using two-tailed Student's *t*-tests.

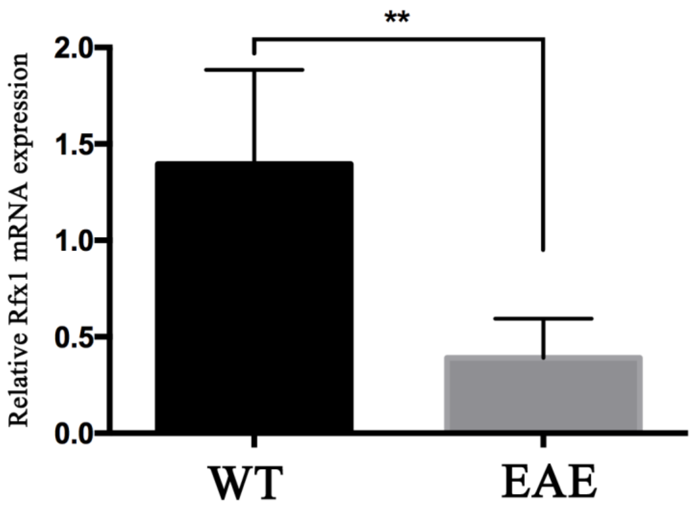

Supplementary Figure 5 Rfx1 expression in $CD4^+$ T cells of EAE mice. qPCR analysis of the relative mRNA expression level of Rfx1 in $CD4^+$ T cells from EAE mice on day 19 and wild-type (WT) mice. Data are representative of two independent experiments (mean ± s.d.; n=5). **P<0.01, compared with the indicated groups. P-values were determined using two-tailed Student's *t*-tests.

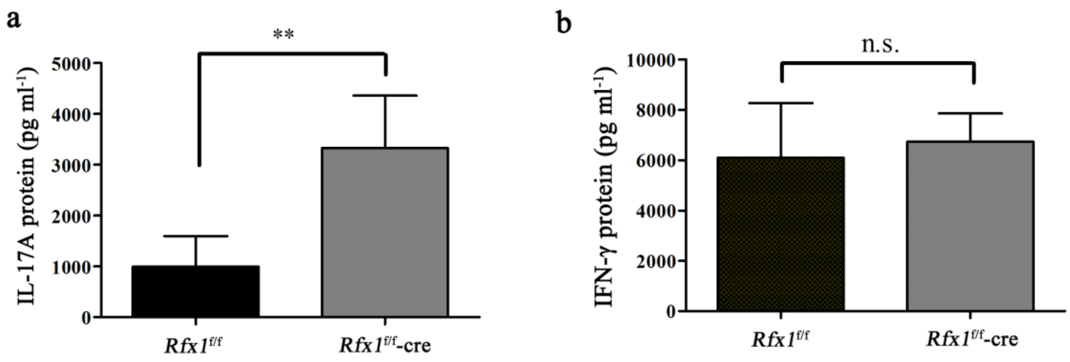

Supplementary Figure 6 Secretion of IL-17A and IFN-γ proteins from splenocyte cells with MOG35-55 re-stimulation. Splenocyte were isolated from spleen of *Rfx1*$^{f/f}$ and *Rfx1*$^{f/f-}$cre mice immunized with MOG35-55 at day 19 and further cultured in vitro with MOG35-55 (30 μg) for 3 days. IL-17A **(a)** and IFN-γ **(b)** concentrations in culture supernatant were measured by ELISA. Data are representative of two independent experiments (mean ± s.d.; n=5). **P<0.01; n.s., not significant, two-tailed Student's *t*-test.

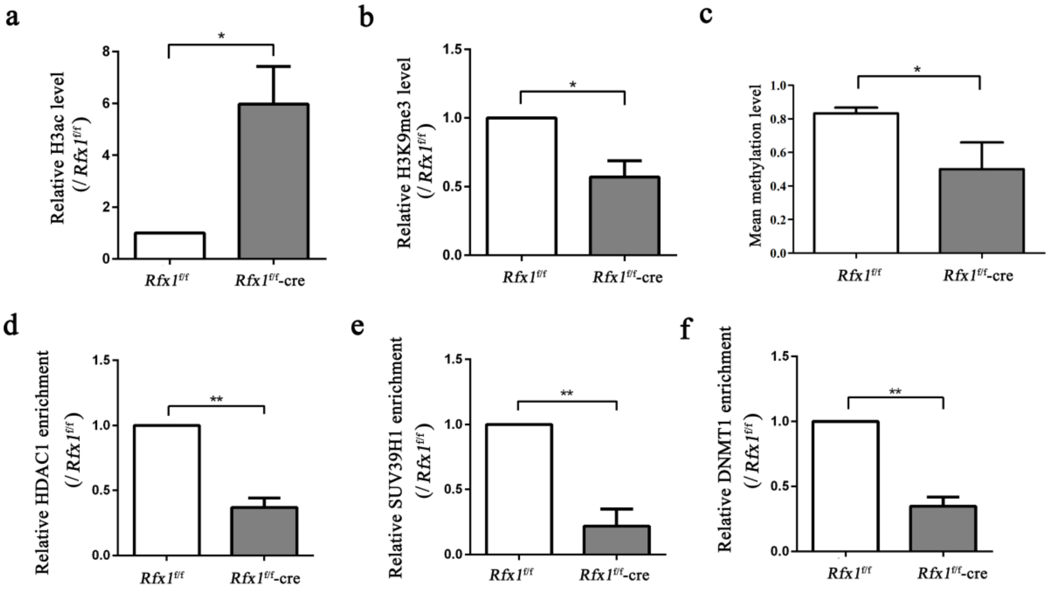

Supplementary Figure 7 The levels of histone H3 acetylation, H3K9 tri-methylation and the enrichment of HDAC1, SUV39H1 and DNMT1 in the promoter of *Il17a* in conditional knockout mice. (a, b) ChIP-qPCR analysis of the levels of H3 acetylation **(a)** and H3K9me3 **(b)** in the promoter of *Il17a* in $CD4^+$ T cells of *Rfx1*$^{f/f}$ and *Rfx1*$^{f/f}$-cre mice. **(c)** the mean methylation levels of 4 CG pairs in mouse *Il17a* promoter (217bp, 201bp, 117bp and 90bp upstream of transcription start site) in $CD4^+$ T cells of *Rfx1*$^{f/f}$ and *Rfx1*$^{f/f}$-cre mice. **(d-f)** ChIP-qPCR analysis of the enrichment of HDAC1 **(d)**, SUV39H1 **(e)** and DNMT1 **(f)** in the promoter of *Il17a* in $CD4^+$ T cells of *Rfx1*$^{f/f}$ and *Rfx1*$^{f/f}$-cre mice. The levels in *Rfx1*$^{f/f}$ groups were set to "1". The fold-changes were calculated relative to the *Rfx1*$^{f/f}$ groups. Data are representative of three independent experiments (mean ± s.d.; n=3). *P<0.05 and **P<0.01, compared between the indicated groups. P-values were determined using two-tailed Student's *t*-tests.

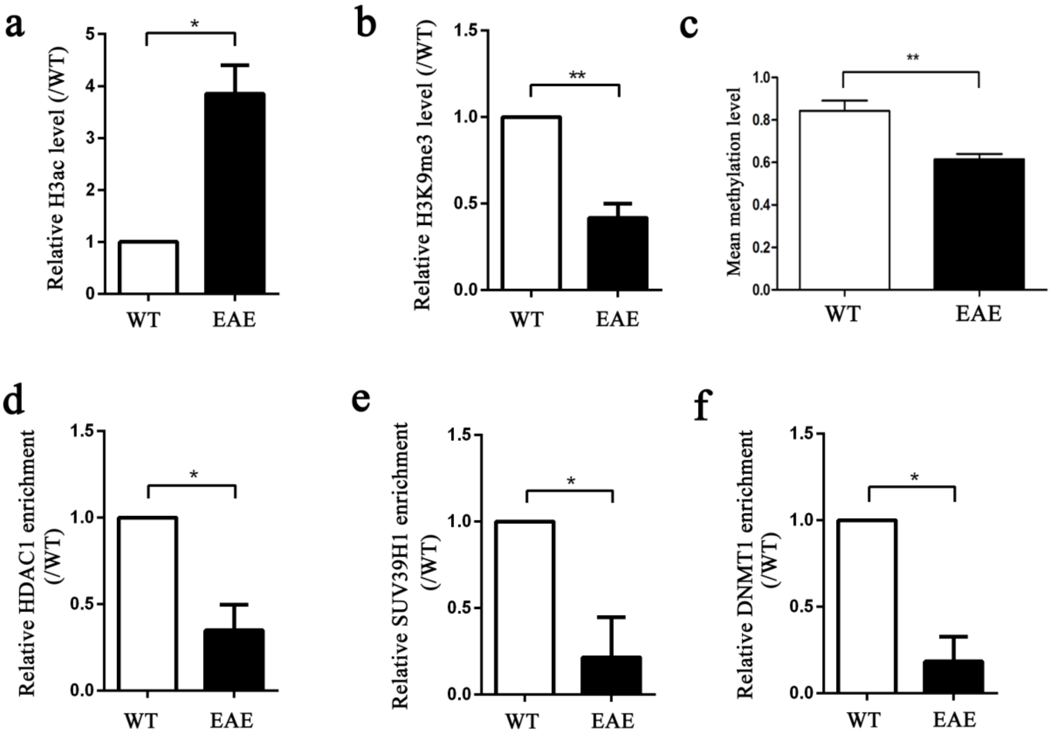

Supplementary Figure 8 The levels of histone H3 acetylation, H3K9 tri-methylation and the enrichment of HDAC1, SUV39H1 and DNMT1 in the *Il17a* promoter in EAE mice. (a, b) ChIP-qPCR analysis of the levels of H3 acetylation **(a)** and H3K9me3 **(b)** in the promoter of *Il17a* in CD4$^+$ T cells of EAE mice on day 19 and wild-type mice. **(c-e)** ChIP-qPCR analysis of the enrichment of HDAC1 **(d)**, SUV39H1 **(e)** and DNMT1 **(f)** in the promoter of *Il17a* in CD4$^+$ T cells of EAE mice on day 19 and wild-type (WT) mice. The levels in WT groups were set to "1". The fold-changes were calculated relative to WT groups. Data are representative of three independent experiments (mean ± s.d.; n=3). *P<0.05 and **P<0.01, compared between the indicated groups. P-values were determined using two-tailed Student's *t*-tests.

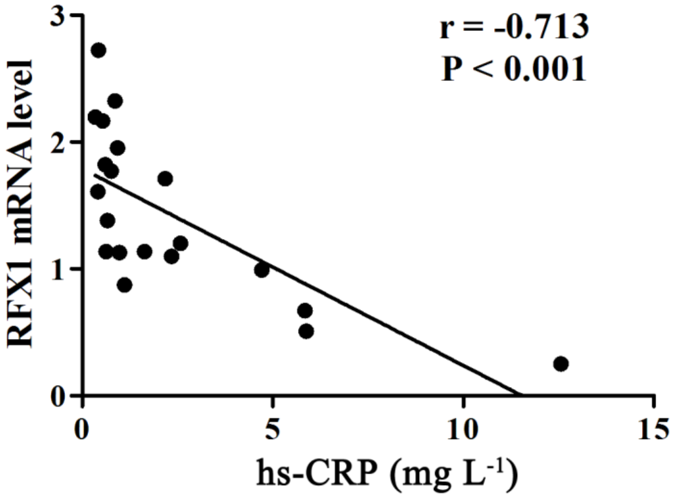

Supplementary Figure 9 The correlation between RFX1 expression levels and serum CRP levels. RFX1 mRNA expression levels in $CD4^+$ T cells and high-sensitivity CRP levels in serum were measured in SLE patients with active arthritis (n=20). Pearson's correlation coefficient was used for the correlation analysis (two-tailed).

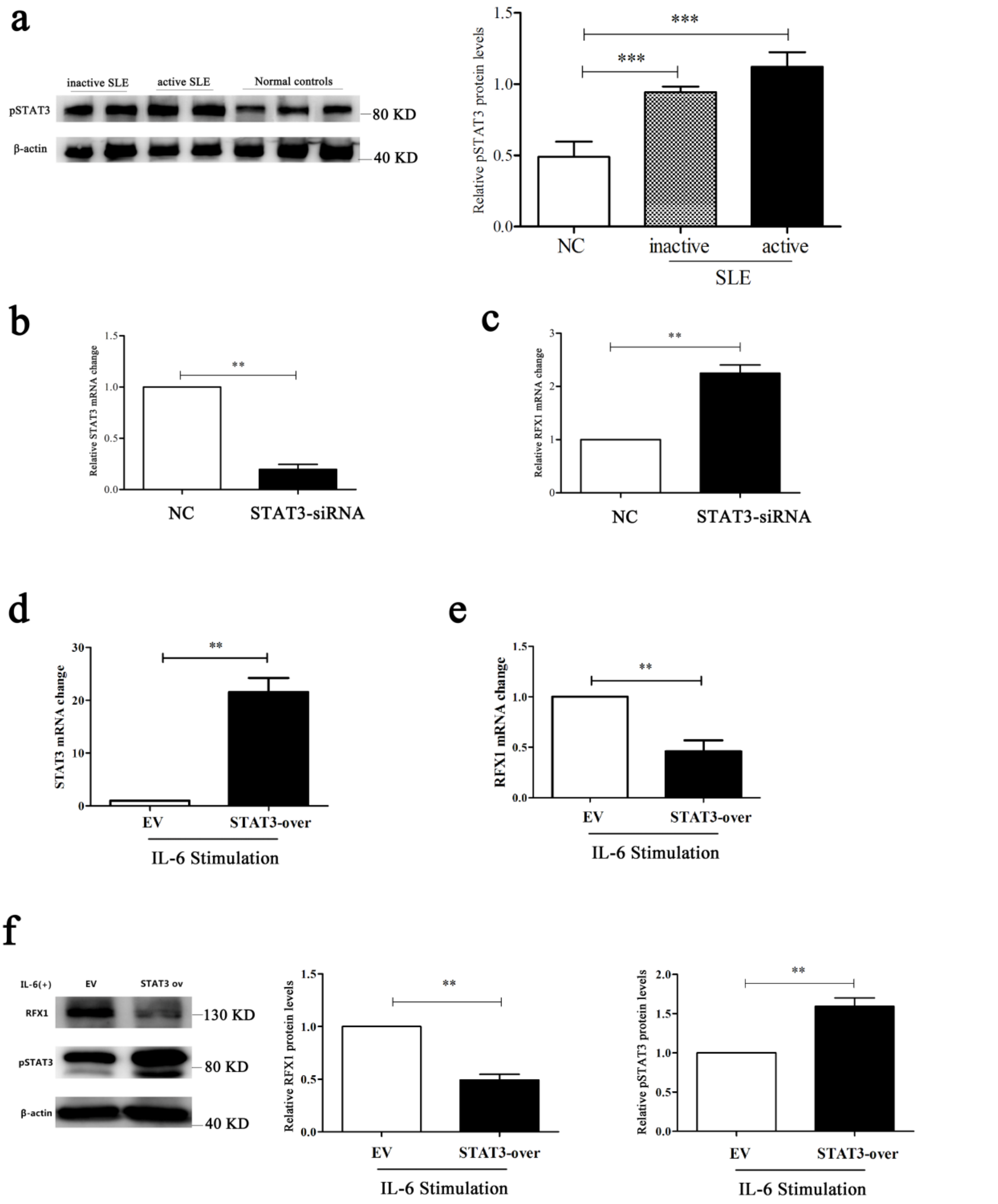

Supplementary Figure 10 STAT3 regulates RFX1 expression. (a) Western blot analysis of phosphorylated STAT3 (pSTAT3) protein levels in $CD4^+$ T cells of inactive (n=8) and active (n=16) SLE patients and healthy controls (n=12). **(b, c)** RT-qPCR analysis of STAT3 and RFX1 mRNA expression in $CD4^+$ T cells transfected with STAT3 siRNA or negative control. **(d, e)** RT-qPCR analysis of STAT3 and RFX1 mRNA expression in $CD4^+$ T cells transfected with STAT3 expression vector (STAT3-over) or empty control vector (EV) under IL-6 stimulation. **(f)** changes of pSTAT3 and RFX1 proteins in $CD4^+$ T cells transfected with STAT3 expression vector (STAT3-over) or empty control vector (EV) under IL-6 stimulation. Data are representative of three independent experiments (mean ± s.d.; n=3; **b-f**). *P<0.05, **P<0.01 and ***P<0.001, compared between the indicated groups. P-values were determined using two-tailed Student's *t*-tests.

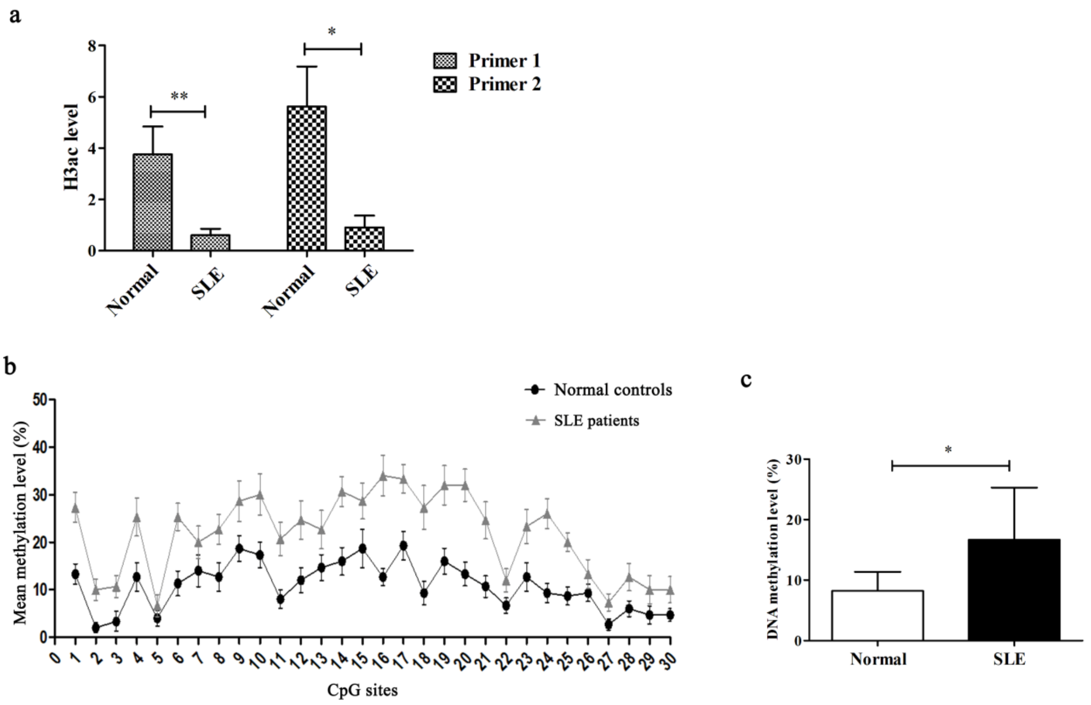

Supplementary Figure 11 Histone acetylation levels and DNA methylation levels in intron 7 of the *RFX1* gene. (a) ChIP-qPCR analysis of H3 acetylation levels of intron 7 in $CD4^+$ T cells of SLE patients (n=8) and normal controls (n=8). **(b, c)** BSP analysis of DNA methylation levels of the CpG island in intron 7 in $CD4^+$ T cells of SLE patients (n=15) and healthy controls (n=15). The methylation level of each CpG site **(b)** and mean methylation levels of all CpG sites **(c)**. Data are mean ± s.d. *P<0.05 and **P<0.01, compared between the indicated groups. P-values were determined using two-tailed Student's *t*-tests.

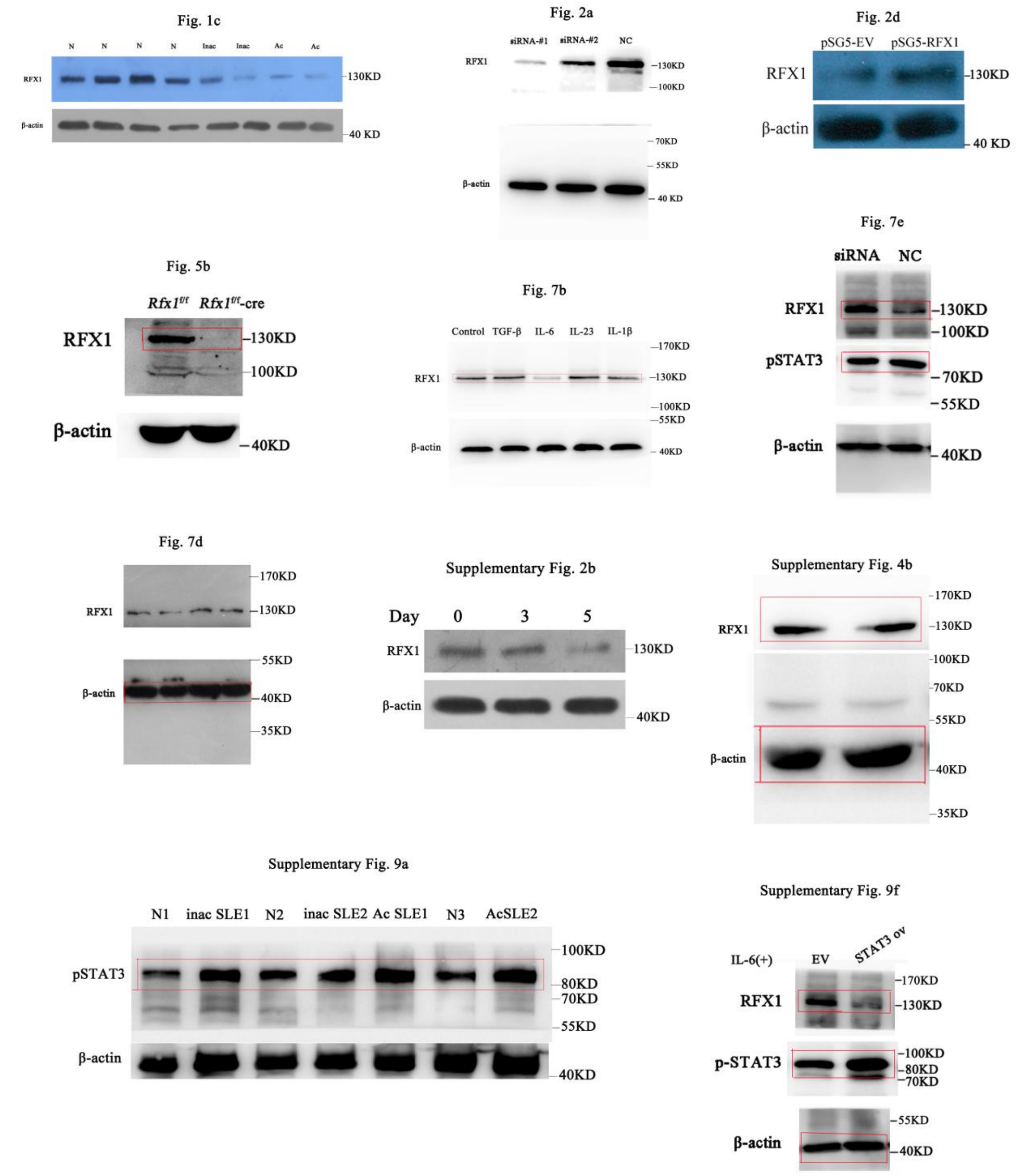

Supplementary Figure 12 Uncropped scans of blots.

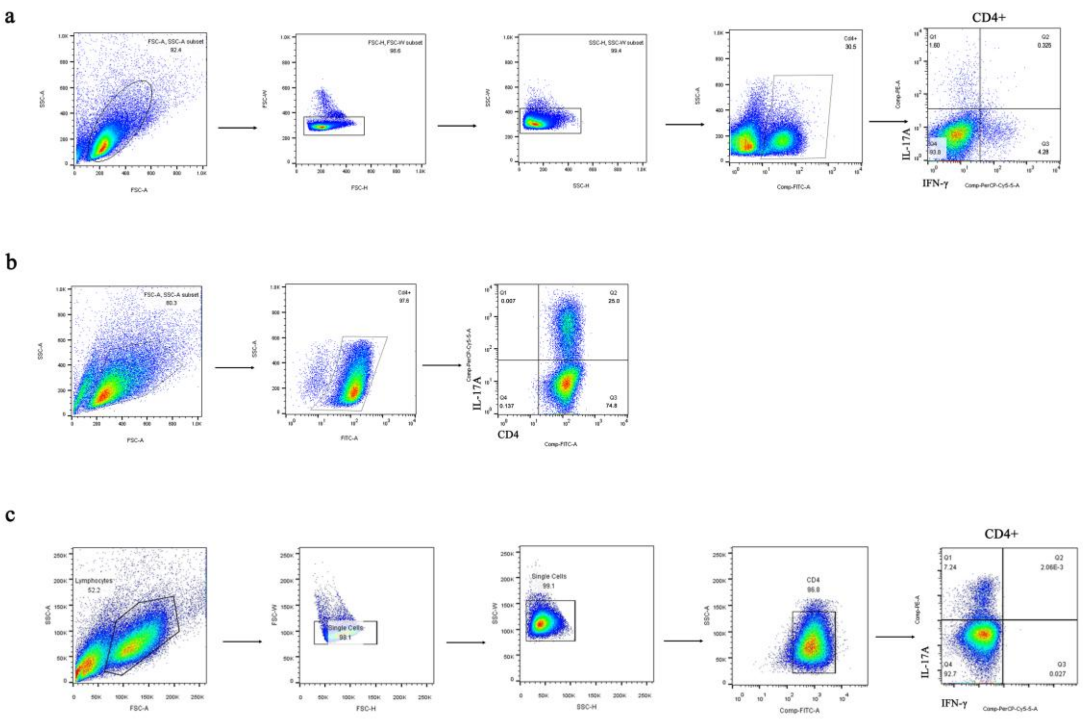

Supplementary figure 13 Flow Cytometry gating strategies. (a) Murine splenocytes and lymph nodes stained for CD4, IL-17A and IFN-γ as shown in Figure 5e and Figure 7g. **(b)** Murine Th17 cells induced in vitro stained for CD4 and IL-17A as shown in Figure 6. **(c)** Human Th17 cells induced in vitro stained for CD4 and IL-17A as shown in Supplementary figure 2c.

Supplementary Table 1. Demographic information for SLE patients.

SLE	Age (year)	Sex	SLEDAI*	hs-CRP(mg L^{-1})
lupus1	49	Female	0	/
lupus 2	24	Female	4	/
lupus 3	32	Female	2	/
lupus 4	47	Female	6	/
lupus 5	44	Female	4	/
lupus 6	42	Female	4	/
lupus 7	24	Female	0	/
lupus 8	41	Female	2	/
lupus 9	50	Female	6	/
lupus 10	47	Female	14	/
lupus 11	30	Female	6	/
lupus 12	27	Female	10	/
lupus 13	38	Female	11	/
lupus 14	17	Female	16	/
lupus 15	24	Female	24	/
lupus 16	49	Female	6	/
lupus 17	45	Female	10	/
lupus 18	22	Female	10	/
lupus 19	59	Female	12	/
lupus 20	39	Female	8	/
lupus 21	21	Female	16	/
lupus 22	19	Female	12	/
lupus 23	33	Female	18	/
lupus 24	34	Female	16	/
lupus 25	45	Female	18	/
lupus 26	23	Female	23	/
lupus 27	23	Female	8	/
lupus 28	31	Female	28	/
lupus 29	40	Female	5	/
lupus 30	20	Female	12	/
lupus 31	46	Female	15	/
lupus 32	22	Female	7	/
lupus 33	41	Female	20	/
lupus 34	25	Female	4	/
lupus 35	32	Female	2	/
lupus 36	47	Female	4	/
lupus 37	39	Female	2	/
Lupus38	33	Female	14	/
Lupus39	41	Female	16	/
Lupus40	34	Female	10	/

Lupus41	18	Female	6	/
Lupus42	21	Female	21	/
Lupus43	23	Female	2	/
Lupus44	25	Female	6	/
Lupus45	27	Female	9	/
Lupus46	30	Female	13	/
Lupus47	40	Female	14	/
Lupus48	18	Female	14	/
Lupus49	35	Female	16	/
Lupus50	25	Female	17	/
Lupus51	34	Female	18	/
Lupus52	46	Female	18	/
Lupus53	18	Female	24	/
Lupus54	41	Female	4	/
Lupus55	21	Female	2	/
Lupus56	28	Female	0	/
Lupus57	31	Female	1	/
Lupus58	35	Female	4	/
Lupus59	23	Female	4	/
Lupus60	46	Female	10	/
Lupus61	43	Female	10	0.54
Lupus62	35	Female	12	1.11
Lupus63	16	Female	17	2.58
Lupus64	45	Female	14	0.93
Lupus65	63	Male	11	4.71
Lupus66	52	Female	16	2.35
Lupus67	48	Male	4	0.98
Lupus68	44	Female	10	0.76
Lupus69	31	Female	15	0.35
Lupus70	24	Female	20	5.85
Lupus71	24	Female	27	0.41
Lupus72	41	Female	8	0.63
Lupus73	26	Female	11	1.64
Lupus74	29	Female	4	0.43
Lupus75	23	Female	10	0.6
Lupus76	54	Female	10	0.66
Lupus77	57	Female	6	2.18
Lupus78	43	Female	6	12.56
Lupus79	28	Female	25	0.86
Lupus80	50	Female	19	5.88

* SLEDAI: SLE Disease Activity Index; hs-CRP: high sensitive C-Reactive Protein

Supplementary Table 2. RT-qPCR primers

Gene	species	Forward	Reverse
RFX1	human	GATCCAAGGCGGCTACAT	CAGCCGTCTCATAGTTGTCC
IL17A	human	AATCTCCACCGCAATGAGGACC	TGCTGGATGGGGACAGAGTTCA
IL17F	human	AGTAAGCCACCAGCGCAACATG	CTCAGAAAGGCAAGCCCCAATA
RORC	human	AGGCCATTCAGTACGTGGTGGA	CGTGCGGTTGTCAGCATTGTAG
STAT3	human	GGAGGAGGCATTCGGAAAG	TCGTTGGTGTCACACAGAT
β-actin	human	GAGCTACGAGCTGCCTGACG	GTAGTTTCGTGGATGCCACAG
GAPDH	human	ATGGGGAAGGTGAAGGTCG	GGGGTCATTGATGGCAACAATA
Il17a	mouse	CTCACACGAGGCACAAG	CTCAGCAGCAGCAACAG
Il17f	mouse	GGGAAGAAGCAGCCATTG	TCCAGGGGAGGACAGTT
Rfx1	mouse	GTCAGAAGCCAGCCCCAGTT	CTTACCTGCTGTGGCACCTGAATG
Rorc	mouse	GGACAGGGAGCCAAGTTCTCA	CACAGGTGATAACCCCGTAGTGG
Il23r	mouse	GCAGGAAGTATTTGGTATGGG	GAAATGATGGACGCAGAAGG
β-actin	mouse	CTGAGAGGGAAATCGTGCGT	AACCGCTCGTTGCCAATAGT

Supplementary Table 3. PCR primers for site-directed mutant plasmids.

name	Forward	Reverse
Mu(site1)	ATCCTTCCCCTTTCCCAGCCCCCTGGCAGCTCAGG	GGGGCTGGGAAAGGGGAAGGATGTA
Mu(site2)	GATTCCAAGTTCTGTCCCACCAACCGGGGCCC	GGACAGAACTTGGAATCACTGACAGGGCCTTA
Mu(site3)	TGTGCCCGGTTTCCAAGCCCCACCCCTGAGGCCC	CTTGGAAACCGGGCACAGGATGTGAGGGGCGG

Supplementary Table 4. ChIP-qPCR primers

Gene	species	Forward	Reverse
IL17A-Primer1	human	CTAGTTCTCATCACTCTCTACTCCC	ATTGAATTTAACAATTCTTTTGTTG
IL17A-Primer2	human	TTCATTTTTTGTTTACTTATATGAT	GAGTTATGCTCTATTTTAATGGTTC
IL17A-Primer3	human	TCCTGGAGCATGGTGGGGGGTAAGG	TTCTTCTGCCATTAGCTTGCATACA
RFX1-Primer1	human	CAGACACTGCCCTACATCCTTC	TCGGAGAGGGGTTAGAACTGAC
RFX1-Primer2	human	TGATTCCAGGTTCTGTCCCACC	GGAGCTACAGAAAGGCCTCAG
Il17a	mouse	GAGTGGGTTTCTTTGGGCAA	AGCATGACTTCTTGGGAGCT

Supplementary Table 5. BSP primers

Gene	species	Forward	Reverse
IL17A	human	Outer: TGGTTAAGGAATTTGTGAGGA	Outer: TCTCCATAATCAAAACCCAAC
		Inter: AATTTTTGTTTTTTTTATTTTTTTT	Inter: AAAACTCACCACCAATAAAATCTTC
RFX1	human	Outer: GGTTTTGGGTTAGTTTTAATTTTT	Outer: TTCTCTAAATCCTAACCCTCTAA
		Inter: GGTGGAGGTTTGGAGTTT	Inter: ACAAAAACAAATATAAAAACAACA
Il17a	mouse	Outer: AGTTAGGGAATTTGGTAGAAAAGT	Outer: AGTGCAGGACTCACCACAGA
		Inter: AAGTGTGTGTTATTAGGAGATTGT	Inter: ATGAAGCTCTCCCTAAACTCA
